# Editorial: Exposure science and occupational health: insights from ISES 2022

**DOI:** 10.3389/fpubh.2024.1515173

**Published:** 2024-11-21

**Authors:** Marina Almeida-Silva, Susana Viegas, Brian Curwin, M. Elizabeth Marder, Urs Schlüter

**Affiliations:** ^1^H&TRC-Health & Technology Research Center, ESTeSL-Escola Superior de Tecnologia da Saúde, Instituto Politécnico de Lisboa, Lisbon, Portugal; ^2^OSEAN-Outermost Regions Sustainable Ecosystem for Entrepreneurship and Innovation, Funchal, Portugal; ^3^Public Health Research Centre, Comprehensive Health Research Center, CHRC, REAL, CCAL, NOVA National School of Public Health, NOVA University Lisbon, Lisbon, Portugal; ^4^National Institute for Occupational Safety and Health, Centers for Disease Control and Prevention (CDC), Cincinnati, OH, United States; ^5^California Department of Public Health, Sacramento, CA, United States; ^6^Unit 4.I.4 Exposure Assessment, Exposure Science, Division 4 Hazardous Substances and Biological Agents, Federal Institute for Occupational Safety and Health (BAuA), Dortmund, Germany

**Keywords:** occupational exposure, workplace exposure, exposure science, modeling, biomonitoring, inhalation exposure, dermal exposure, occupational health and safety

## Introduction

The annual meeting of the International Society of Exposure Science – from exposure to human health: new developments and challenges in a changing environment – took place from 25 to 29 September 2022 in Lisbon, Portugal. The aim of the conference was to promote information sharing and facilitate discussion on exposure sciences and related fields in the context of the changing environment, especially how we – as exposure scientists - can better understand and respond to the complex and multidisciplinary issues in exposure and environmental health through sciences and policies. This research topic now presents insights on exposure science and occupational health that were presented during the ISES 2022 conference covering all aspects of occupational exposure science. The ISES 2022 conference resulted in many abstracts being submitted (more than 450 submissions) describing the findings of new research on exposure science. The final participation numbers were 421 in-person attendees and 35 virtual attendees. It should be mentioned that 93 (20%) attendees were students or new researchers. The main geographical origins of our attendees were North America (42%) followed by Europe (39%).

Modern exposure science is rooted in the industrial hygiene and radiation health physics practices of the last century, and exposure science continues to play an important role in occupational health. Today, an individual may encounter a wide range of agents that directly or indirectly result in some form of adverse effect or harm. Generally referred to as “stressors,” these agents can be chemical, physical, biological, or psychosocial, as well as mixtures thereof. Exposure science is the distinct discipline that encompasses the study of receptors and their behaviors related to contact with such stressors, the nature and extent of such contact, and the fate of these stressors over space and time.

The scientific articles published under the scope of this Research Topic “*Exposure Science and Occupational Health: Insights from ISES 2022*” cover various aspects of occupational and environmental exposures.

In recent research spanning from small business beauty salons in Arizona to hospitals in the Czech Republic and Slovakia underscores the urgent need for stricter workplace safety measures (Bláhová et al.; Ramírez et al.). These studies offer valuable insights into how chemicals used in everyday occupational tasks—ranging from antineoplastic drugs in hospitals to hair products in salons—pose serious risks to human health. Despite this, regulatory frameworks and practical protective guidelines are often fragmented or outdated.

One particularly poignant study examines the contamination of surfaces in hospitals and pharmacies by antineoplastic drugs (ADs) commonly used in cancer treatment. These drugs are essential for patient care but represent a substantial risk to healthcare workers due to their carcinogenic and mutagenic properties (Bláhová et al.). The study analyzed over 2,200 samples in healthcare facilities and recommended technical guidance values (TGVs) for managing this contamination. This is a crucial step toward minimizing the occupational exposure of healthcare personnel, but the broader challenge remains—how do we balance life-saving drugs with workplace safety? The authors proposed setting contamination limits at 100 pg/cm^2^ in most healthcare settings, but the complexity of these environments means that even low-level contamination in areas like staff rooms can lead to unintended exposure. The “no-threshold effects” of genotoxic drugs complicate establishing safe exposure limits, making prevention and meticulous monitoring indispensable. Here, prevention might take the form of enforcing cleaning protocols and limiting access to highly contaminated areas.

Equally concerning is the exposure of workers in beauty salons to VOCs, as highlighted in a study from Tucson, Arizona (Ramírez et al., also Lothrop et al. “*Studying full-shift inhalation exposures to volatile organic compounds (VOCs) among Latino workers in very small-sized beauty salons and auto repair shops*”). Beauty salons, a multibillion-dollar global industry, expose workers to harmful chemicals found in hair and beauty products, which volatilize and contaminate the air during routine tasks such as hair styling or nail treatments. This research emphasizes how salon workers—largely women of color with limited health insurance—bear the brunt of these exposures, which are often higher than for the average population.

This study found that VOC exposure levels vary significantly between salons due to differences in ventilation, product usage, and services offered. Salon workers frequently experience reproductive issues, respiratory problems, and skin disorders, yet few regulations govern these settings. Like the hospital contamination study, this research underscores the need for stronger regulatory action, particularly as these exposures can result in long-term health issues.

In another example of chemical exposure, a study on neonicotinoid insecticides—commonly used in household settings on plants and pets—illustrates how pervasive chemical exposure is in modern life (Wrobel et al.). This research focuses on two widely used insecticides, acetamiprid and imidacloprid, and their presence in the urine of volunteers after household use. Although the study concludes that exposure levels are well below acceptable limits, it highlights the importance of biomonitoring to detect and manage human exposure to hazardous chemicals. Regular users of these chemicals, such as professional gardeners or pet care workers, are particularly vulnerable to cumulative exposure. As the study suggests, more detailed research is needed to fully assess the risks to those who use these chemicals regularly in professional context.

Also, fields that are typically understudied receive attention. The publication by Dietz et al. evaluates systematically the scientific literature about the relevance of oral exposure in workplaces concluding that oral exposure is considered as potentially contributing (123 studies) or explicitly relevant (80 studies). The exposure of firefighters at fire training facilities and of employees at respiratory protection and hose workshops was examined by biomonitoring (Koslitz et al.). Polycyclic aromatic hydrocarbons were found in a fivefold increase in mean for firefighters. However, levels in workshop employees were found to be low, with the majority of urine samples yielding concentrations below the limit of quantification.

Different methods of exposure assessment are reported. Exposure modeling was used in two studies (Aachimi et al.; Hahn et al.), biomonitoring is reported in two studies (Koslitz et al.; Wrobel et al.) and workplace air monitoring is reported in three studies (Sabic et al.; Lothrop et al.; Ramírez et al.). Most of the studies evaluated occupational exposure to different chemicals. However, also microbiological contamination at workplaces is described (Viegas, Eriksen et al.; Viegas, Dias et al.).

The studies on spray processes (Sabic et al.; Hahn et al.), including the use of volatile solvents in industrial painting, bring another layer to the occupational exposure discussion. Real-time monitoring of solvent evaporation during spray application and drying processes showed that secondary exposure during drying often exceeded the initial exposure from spraying. This finding has significant implications for industries like manufacturing and construction, where workers may not realize that drying paints or solvents continue to release harmful chemicals into the air long after application. These studies call for the refinement of existing exposure models and the development of better predictive tools for workplace safety. For example, the current models used for estimating airborne concentrations during spray processes often overestimate or underestimate actual exposure levels. Enhanced, real-time data collection and more accurate exposure models are essential to creating safer work environments.

These studies collectively raise awareness about the silent dangers posed by everyday exposures at workplaces, offering a clarion call for stronger policies, better monitoring systems, and above all, a commitment to worker safety across all industries.

